# Association of Infant Physical Development and Rapid Growth With Pubertal Onset Among Girls in Rural China

**DOI:** 10.1001/jamanetworkopen.2021.6831

**Published:** 2021-05-03

**Authors:** Jing Wei, Shuang Liu, Yue Cheng, Wenfang Yang, Zhonghai Zhu, Lingxia Zeng

**Affiliations:** 1 Department of Epidemiology and Biostatistics, School of Public Health, Xi’an Jiaotong University Health Science Center, Xi’an, China; 2 Department of Nutrition and Food Safety Research, School of Public Health, Xi’an Jiaotong University Health Science Center, Xi’an, China; 3 Department of Obstetrics and Gynecology, Maternal and Child Health Center, The First Affiliated Hospital of Health Science Center in Xi’an Jiaotong University, Xi’an, China; 4 Key Laboratory of Environment and Genes Related to Diseases, Xi’an Jiaotong University, Ministry of Education, Xi’an, China

## Abstract

**Importance:**

Whether physical development and rapid growth during the first 2 years of life are associated with puberty onset in girls remains unclear.

**Objective:**

To examine the associations of infant physical development and rapid growth with the timing of puberty onset in girls.

**Design, Setting, and Participants:**

This prospective birth cohort study included girls whose mothers had participated in a randomized, double-blind cluster trial of micronutrient supplementation with a daily capsule of folic acid, iron and folic acid, or multiple micronutrients during pregnancy. A total of 547 singleton girls in rural western China who were born after 2004 were followed up at 3, 6, 12, and 24 months of age and were eligible for long-term follow-up. Of these, 294 adolescent girls (aged 10-12 years), representing 53.7% of the total sample, were included in the follow-up interview that was conducted from June 1 through December 31, 2016. Data analyses were conducted from November 1, 2019, to May 30, 2020.

**Exposures:**

Infant weight and length were assessed at birth and 3, 6, 12, and 24 months of age. Rapid growth, defined as a weight-for-age or length-for-age *z* score increase of greater than 0.67, was calculated during 5 periods: birth to 3 months, birth to 6 months, 6 to 12 months, 12 to 24 months, and birth to 24 months of age.

**Main Outcomes and Measures:**

Puberty stage was assessed using the standardized Tanner staging system. Onset of puberty was defined as attaining Tanner stage 2 of breast development or pubic hair growth, and the corresponding age at follow-up was recorded.

**Results:**

Of 294 girls included in the analysis (mean [SD] age, 11.25 [0.57] years), the median age of puberty onset was 11 (interquartile range, 10-11) years. A significant association with puberty onset was found for infancy weight-for-age *z* score at 12 months of age (adjusted hazard ratio [HR], 1.20; 95% CI, 1.01-1.44) and rapid weight gain from birth to 24 months of age (adjusted HR, 1.40; 95% CI, 1.01-1.93) and birth to 3 months of age (adjusted HR, 1.39; 95% CI, 1.02-1.91). However, length-related indicators during early life were not associated with adolescent puberty onset.

**Conclusions and Relevance:**

This study found that a higher weight-for-age *z* score and rapid weight gain during infancy, particularly from birth to 3 months of age, were associated with an increased likelihood of puberty onset in girls. These findings suggest that weight-related indicators may serve as proxies of puberty onset among adolescent girls.

## Key Points

**Question:**

What is the long-term association of infant physical development and rapid growth with the timing of puberty onset in girls?

**Findings:**

In this birth cohort of 294 adolescent girls in rural China, the infancy weight-for-age *z* score at 12 months of age and rapid weight gain from birth to 24 months of age and birth to 3 months of age were associated with puberty onset.

**Meaning:**

The findings suggest that weight-related indicators may serve as proxies of puberty onset among adolescent girls.

## Teaser

This birth cohort study examines the associations of infant physical development and rapid growth with the timing of puberty onset in girls in China.

## Introduction

Growing evidence indicates that puberty onset has been occurring at an earlier age in girls.^1,2^ This may increase the risk of diseases in adulthood, such as breast, ovarian, and endometrial cancers, metabolic syndrome, and cardiovascular disease.^3,4^ A worldwide meta-analysis^1^ concluded that age at puberty onset among girls has decreased by approximately 3 months per decade from 1977 to 2013.

The literature to date has largely focused on secular increases in body mass index (BMI) in early childhood and their associations with the timing of puberty (ie, menarche and puberty onset),^5^ with higher BMI in childhood associated with earlier puberty.^6,7^ It remains unclear whether body size during the first 2 years of life, a critical window that lays the foundation for long-term health outcomes, is also associated with puberty. A systematic review based on cohort studies^8^ reported that small for gestational age (SGA) and low birth weight (LBW) were associated with earlier age at puberty onset in girls (weighted mean difference, 0.64; 95% CI, 1.21-0.06). Few studies have detailed measures of weight and height during childhood,^9^ and these studies lacked specific secular data during the first 2 years of life. Infant rapid growth, compensating for intrauterine growth restriction, is usually observed during the first 2 years after birth.^10^ Emerging evidence suggests that the timing of rapid growth (tempo) itself may play an independent role in modifying subsequent health outcomes.^11^ However, it is not clear which period of rapid growth during infancy results in earlier puberty. Few studies have examined the association of rapid weight gain in infancy with puberty, and limited assessments have been performed.^12,13^ The DONALD (Dortmund Nutritional and Anthropometric Longitudinally Designed) Study from Germany^12^ assessed rapid weight gain with measurements at birth and at 24 months of age. Another birth cohort from South Africa focused on transient rapid weight gain from birth to 12 months of age.^13^ Thus, a study with multiple prospective measures of growth during the first 2 years of life is needed to determine the specific period associated with the timing of puberty.

To assess puberty onset, previous studies^9,14^ have mainly used the timing of menarche, a relatively late milestone of pubertal development, which may mask the true associations of infant growth in early life with puberty onset. In addition, growth during early life may interfere with the onset of puberty but not the subsequent stages or durations (ie, the timing of menarche).^12^

In the present study, we aimed to investigate the associations of infant physical development and rapid growth during early life with puberty onset in girls. We used birth cohort data in rural western China, with physical development (weight, length, and rapid growth) assessed at birth and at 3, 6, 12, and 24 months of age and puberty assessment at early adolescence (10-12 years of age). Our results may help to identify the strongest indicator of physical development that is associated with puberty onset and its critical period within the first 2 years after birth.

## Methods

### Study Design and Participants

The study design was a prospective birth cohort of participants whose mothers participated in a randomized, double-blind cluster trial of micronutrient supplementation in pregnancy.^15^ The parent trial is detailed elsewhere.^16^ Briefly, pregnant women in the designated villages were randomly allocated to daily supplementation with folic acid (400 μg [control group]), iron (60 mg) and folic acid (400 μg), or multiple micronutrients in accordance with the World Health Organization’s recommended allowance of 15 vitamins and minerals; supplements were used from enrollment to delivery.^17^ In the trial, 4604 singleton births occurred, but only 1400 births in 2004-2006 were enrolled in the long-term follow-up cohort. A total of 1388 births were followed up after excluding deaths (n = 3), children with birth defects (n = 7), and parents with disabilities (n = 2). Among them, 547 girls were eligible for long-term follow-up. Finally, we tracked 294 girls (53.7%) from birth through 3, 6, 12, and 24 months of age and into early adolescence (10-12 years of age). Follow-up data were collected from June 1 through December 31, 2016.

The follow-up evaluations were approved by the ethics committee of the Xi’an Jiaotong University Health Science Center in Xi’an, China. Parents or caregivers provided signed informed consent, and verbal assent was obtained from each child. This study followed the Strengthening the Reporting of Observational Studies in Epidemiology (STROBE) reporting guideline.

### Anthropometric Measurements

#### Weight and Linear Growth

Birth weight and length were measured by the nursing staff within 1 hour of delivery at the hospital (263 [89.5%]) or by township maternal and child health care staff within 72 hours for home deliveries (31 [10.5%]). Weight and length at 3, 6, 12, and 24 months of age at each follow-up visit were measured using standard World Health Organization procedures. Weight was measured to the nearest 10 g using an electronic scale (type BD-585; Tanita Corporation) after removing heavy clothes. Length was measured to the nearest 1 mm using a portable measuring board with a fixed headpiece.

Low birth weight was defined as less than 2500 g. Body mass index was calculated as weight in kilograms divided by the square of body height in meters. The infant weight-for-age *z* score (WAZ), length-for-age *z* score (LAZ), and BMI-for-age *z* score (BAZ) were calculated using the World Health Organization Child Growth Standards 2006.^18^ The birth WAZ and LAZ were accordingly calculated by International Fetal and Newborn Growth Consortium for the 21st Century (INTERGROWTH-21st) standards.^19^ Infant stunting was defined as an LAZ less than −2.00 SD; underweight, an WAZ less than −1.00 SD; and overweight, a BAZ greater than 2.00 SD.^20,21^

Changes in *z* scores among infants were calculated using WAZ or LAZ at the end minus the scores at the beginning of every interval of interest, including birth to 3 months of age, birth to 6 months of age, 6 to 12 months of age, 12 to 24 months of age, and birth to 24 months of age. We defined rapid growth as a change in *z* score that was greater than 0.67.^22^

#### Pubertal Development

Trained public health postgraduate students (including Z.Z.) measured the staging of breast development using observation and palpation according to the Tanner staging system, which represents the 5 Tanner stages for breast size. Similarly, the development of pubic hair was staged by visual inspection. We defined the onset of puberty as attaining Tanner stage 2 of breast development or pubic hair growth.^23,24^ Among girls with a Tanner stage greater than 2 (68 [23.1%]), the age of puberty onset was determined by asking the following questions: (1) Have you noticed that your breasts/pubic hair have begun to grow? (2) If yes, how old were you when this began? As for other girls with a Tanner stage of 2 (173 [58.8%]), we used the chronological age at visit as the age of puberty onset.

#### Covariables

Covariables were collected using a structured questionnaire at the enrollment of the parent trial, including sociodemographic characteristics, perinatal factors, and birth outcomes. Sociodemographic characteristics included parental occupation (farmer and others), age at enrollment, and educational attainment (<3 years, primary, secondary, and high school or greater) and household wealth index. Perinatal factors—including maternal parity, self-reported age at menarche, and BMI during the first trimester with maternal weight and height—were measured at the clinic by trained maternal and child health staff, and participants were randomly assigned to a micronutrient regimen (folic acid, iron and folic acid, or multiple micronutrients). Birth outcomes included SGA, which was defined as birth weight less than the 10th population percentile according to the INTERGROWTH-21st standard.^19^ The household wealth index was constructed by assessing 17 different household assets or facilities (eg, bicycles, television/video compact disc devices, and washing machines) using the principal components method and was then categorized into tertiles indicating low, middle, and high levels of household wealth.

### Statistical Analyses

Data were analyzed from November 1, 2019, to May 30, 2020. Continuous variables were described using mean (SD) or median (interquartile range [IQR]); categorical variables, frequencies or proportions. Baseline characteristics by puberty onset were compared using χ^2^ tests or analysis of variance. Cox proportional hazard regression models were used to examine the associations of infant growth and rapid growth at specified periods with puberty onset with hazard ratios (HRs) and 95% CIs. Event times were defined as the age of puberty onset. The possible covariables we considered were parental age, parental educational level, parental occupation, household wealth index at enrollment, parity, SGA outcome at birth, maternal age at menarche, maternal BMI, and randomly assigned regimens. Statistical significance was set at a 2-sided *P* < .05. All analyses were conducted with STATA software, version 15 (StataCorp LLC).

## Results

### Baseline Characteristics

Table 1 presents background characteristics by puberty onset. Among the 294 girls included in the analysis with available data (mean [SD] age, 11.25 [0.57] years), 14 (4.8%) were born LBW; 7 (2.4%), were preterm; and 50 (17.5%) were SGA. The median age at adolescence was 11 (IQR, 11-12) years. A total of 241 girls (82.0%) experienced puberty onset, with a median age of 11 (IQR, 10-11) years. In addition, most characteristics were balanced between participants who were followed up and those who were lost to follow-up (eTable in the Supplement). The eFigure in the Supplement shows the flowchart of the participants.

**Table 1.** Background Characteristics of Girls by Puberty Onset in Rural Western China (N = 294)^a^

Characteristic	All (N = 294)	Onset of puberty	
		Yes (n = 241)	No (n = 53)
**Parental during pregnancy**			
Maternal age, mean (SD), y	23.43 (4.09)	23.51 (4.12)	23.08 (3.97)
Maternal educational attainment			
<3 y	12 (4.1)	7 (58.3)	5 (41.7)
Primary	59 (20.1)	50 (84.7)	9 (15.3)
Secondary	168 (57.3)	139 (82.7)	29 (17.3)
High school or higher	54 (18.4)	45 (83.3)	9 (16.7)
Maternal occupation			
Farmer	237 (80.9)	194 (81.9)	43 (18.1)
Other	56 (19.1)	46 (82.1)	10 (17.9)
Parity			
0	231 (78.6)	186 (80.5)	45 (19.5)
1	49 (16.7)	43 (87.8)	6 (12.2)
≥2	14 (4.8)	12 (85.7)	2 (14.3)
Maternal age at menarche, mean (SD), y	15.23 (1.37)	15.17 (1.39)	15.46 (1.26)
Maternal BMI, mean (SD)	20.56 (2.04)	20.57 (2.04)	20.50 (2.03)
Supplement regimen			
Folic acid	99 (33.7)	76 (76.8)	23 (23.2)
Iron and folic acid	94 (32.0)	78 (83.0)	16 (17.0)
Multiple micronutrients	101 (34.4)	87 (86.1)	14 (13.9)
Paternal age, mean (SD), y	26.71 (3.96)	26.73 (4.07)	26.58 (3.42)
Paternal educational attainment			
<3 y	3 (1.0)	3 (100)	0
Primary	21 (7.1)	19 (90.5)	2 (9.5)
Secondary	190 (64.6)	150 (78.9)	40 (21.1)
High school or higher	80 (27.2)	69 (86.3)	11 (13.8)
Paternal occupation			
Farmer	207 (70.5)	172 (83.1)	35 (16.9)
Other	87 (29.6)	69 (79.3)	18 (20.7)
Household wealth index			
Low	71 (24.1)	59 (83.1)	12 (16.9)
Middle	110 (37.4)	82 (74.5)	28 (25.5)
High	113 (38.4)	100 (88.5)	13 (11.5)
**Infant at birth**			
Birth weight, mean (SD), g	3094 (380)	3112 (368)	3013 (426)
Birth length, mean (SD), cm	48.9 (2.3)	48.9 (2.3)	48.7 (2.3)
Gestational age at delivery, mean (SD), wk	40 (1)	40 (1)	40 (2)
SGA (<10th population percentile)			
No	236 (82.5)	198 (83.9)	38 (16.1)
Yes	50 (17.5)	37 (74.0)	13 (26.0)
LBW (<2500 g)			
No	279 (95.2)	230 (82.4)	49 (17.6)
Yes	14 (4.8)	10 (71.4)	4 (28.6)
Preterm birth (<37 wk)			
No	287 (97.6)	235 (81.9)	52 (18.1)
Yes	7 (2.4)	6 (85.7)	1 (14.3)
**Infant at 12 mo**			
WAZ, mean (SD)	0.33 (0.87)	0.44 (0.81)	−0.19 (0.95)
Underweight (WAZ < −1.00 SD)			
No	245 (93.2)	207 (84.5)	38 (15.5)
Yes	18 (6.8)	9 (50.0)	9 (50.0)
LAZ, mean (SD)	−0.58 (0.96)	−0.48 (0.92)	−1.03 (1.03)
Stunting (LAZ < −2.00 SD)			
No	247 (93.9)	208 (84.2)	39 (15.8)
Yes	16 (6.1)	8 (50.0)	8 (50.0)
BAZ, mean (SD)	0.92 (1.06)	1.00 (1.04)	0.57 (1.12)
Overweight (BAZ >2.00 SD)			
No	222 (84.7)	182 (82.0)	40 (18.0)
Yes	40 (15.3)	34 (85.0)	6 (15.0)
**Child at 24 mo**			
WAZ, mean (SD)	0.18 (0.80)	0.25 (0.78)	−0.19 (0.85)
Underweight (WAZ < −1.00 SD)			
No	236 (92.2)	200 (84.7)	36 (15.3)
Yes	20 (7.8)	12 (60.0)	8 (40.0)
LAZ, mean (SD)	−0.89 (0.93)	−0.80 (0.91)	−1.33 (0.90)
Stunting (LAZ < −2.00 SD)			
No	228 (88.7)	195 (85.5)	33 (14.5)
Yes	29 (11.3)	19 (65.5)	10 (34.5)
BAZ, mean (SD)	1.00 (1.00)	1.02 (0.97)	0.86 (1.13)
Overweight (BAZ >2.00 SD)			
No	218 (85.5)	181 (83.0)	37 (17.0)
Yes	37 (14.5)	31 (83.8)	6 (16.2)
**Early adolescence**			
Age, mean (SD), y	11.25 (0.57)	11.30 (0.58)	11.04 (0.48)
HAZ, mean (SD)	0.11 (1.03)	0.30 (0.97)	−0.74 (0.83)
Stunting (HAZ < −2.00 SD)			
No	285 (97.3)	237 (83.2)	48 (16.8)
Yes	8 (2.7)	3 (37.5)	5 (62.5)
BAZ, mean (SD)	−0.39 (1.09)	−0.19 (1.00)	−1.29 (1.03)
Thinness (BAZ < −2.00 SD)			
No	268 (92.7)	227 (84.7)	41 (15.3)
Yes	21 (7.3)	9 (42.9)	12 (57.1)

Abbreviations: BAZ, BMI-for-age *z* score; BMI, body mass index (calculated as weight in kilograms divided by square of height in meters); HAZ, height-for-age *z* score; LAZ, length-for-age *z* score; LBW, low birth weight; SGA, small for gestational age; WAZ, weight-for-age *z* score.

^a^ Unless otherwise indicated, data are expressed as No. (%) of patients. Data are missing for maternal educational attainment (n = 1), occupation (n = 1), age at menarche (n = 6), and BMI (n = 8); at birth, infant birth weight (n = 1), birth length (n = 17), SGA (n = 8), and LBW (n = 1); at 12 months of age, WAZ (n = 31), underweight (n = 31), LAZ (n = 31), stunting (n = 31), BAZ (n = 32), and overweight (n = 32); at 24 months of age, WAZ (n = 38), underweight (n = 38), LAZ (n = 37), stunting (n = 37), BAZ (n = 39), and overweight (n = 39); and in early adolescence, HAZ (n = 1), stunting (n = 1), BAZ (n = 5), and thinness (n = 5).

### Infant Physical Development and Adolescent Puberty Onset

Table 2 shows that adolescent girls who had higher WAZ during the first 2 years of life were more likely to experience puberty onset. Specifically, after adjusting for covariates, a 1.00-SD increase in WAZ was associated with increased likelihood of puberty onset, with adjusted HRs of 1.15 (95% CI, 0.97-1.37) at 3 months of age, 1.09 (95% CI, 0.91-1.31) at 6 months of age, 1.20 (95% CI, 1.01-1.44) at 12 months of age, and 1.18 (95% CI, 0.97-1.42) at 24 months of age. However, we did not find that LAZ or BAZ was associated with puberty onset among adolescent girls (Table 2). Null associations were also observed for WAZ (adjusted HR, 0.92; 95% CI, 0.77-1.11) and LAZ (adjusted HR, 0.95; 95% CI, 0.84-1.06) at birth (Table 2).

**Table 2.** Association of Physical Development With Puberty Onset Among Adolescent Girls in Rural Western China

Development outcome by age	Participants with puberty onset, No./total No. (%)^a^	Unadjusted				Adjusted^b^	
		HR (95% CI)		*P* value		HR (95% CI)	*P* value
**Weight at critical time points during infancy and childhood**							
Birth							
WAZ per 1.00 SD	239/291 (82.1)	1.00 (0.88-1.14)		.99		0.92 (0.77-1.11)	.41
Underweight (< −1.00 SD)							
No	180/213 (84.5)	1 [Reference]		NA		1 [Reference]	NA
Yes	59/78 (75.6)	0.85 (0.63-1.14)		.28		0.79 (0.50-1.26)	.33
3 mo							
WAZ per 1.00 SD	201/246 (81.7)	1.20 (1.03-1.39)		.02		1.15 (0.97-1.37)	.12
Underweight (< −1.00 SD)							
No	193/229 (84.3)	1 [Reference]		NA		1 [Reference]	NA
Yes	8/17 (47.1)	0.46 (0.23-0.94)		.03		0.49 (0.24-1.03)	.06
6 mo							
WAZ per 1.00 SD	206/251 (82.1)	1.20 (1.03-1.41)		.02		1.09 (0.91-1.31)	.34
Underweight (< −1.00 SD)							
No	200/241 (83.0)	1 [Reference]		NA		1 [Reference]	NA
Yes	6/10 (60.0)	0.91 (0.40-2.04)		.81		1.22 (0.50-2.94)	.66
12 mo							
WAZ per 1.00 SD	216/263 (82.1)	1.28 (1.10-1.49)		.001		1.20 (1.01-1.44)	.04
Underweight (< −1.00 SD)							
No	207/245 (84.5)	1 [Reference]		NA		1 [Reference]	NA
Yes	9/18 (50.0)	0.46 (0.24-0.91)		.02		0.51 (0.25-1.04)	.06
24 mo							
WAZ per 1.00 SD	212/256 (82.8)	1.29 (1.09-1.52)		.003		1.18 (0.97-1.42)	.10
Underweight (< −1.00 SD)							
No	200/236 (84.7)	1 [Reference]		NA		1 [Reference]	NA
Yes	12/20 (60.0)	0.56 (0.31-1.01)		.05		0.61 (0.33-1.15)	.13
**Length at critical time points during infancy and childhood**							
Birth							
LAZ per 1.00 SD	238/289 (82.4)	0.99 (0.90 to 1.09)		.88		0.95 (0.84 to 1.06)	.34
Stunting (< −2.00 SD)							
No	217/260 (83.5)	1 [Reference]		NA		1 [Reference]	NA
Yes	21/29 (72.4)	0.85 (0.54-1.33)		.47		1.16 (0.83-1.62)	.40
3 mo							
LAZ per 1.00 SD	202/248 (81.5)	1.22 (1.05-1.41)		.008		1.14 (0.97-1.35)	.12
Stunting (< −2.00 SD)							
No	187/223 (83.9)	1 [Reference]		NA		1 [Reference]	NA
Yes	15/25 (60.0)	0.62 (0.37-1.05)		.08		0.68 (0.38-1.22)	.20
6 mo							
LAZ per 1.00 SD	207/251 (82.5)		1.11 (0.98-1.26)		.10	1.04 (0.90-1.20)	.61
Stunting (< −2.00 SD)							
No	190/223 (85.2)		1 [Reference]		NA	1 [Reference]	NA
Yes	17/28 (60.7)		0.56 (0.34-0.92)		.02	0.69 (0.41-1.17)	.17
12 mo							
LAZ per 1.00 SD	216/263 (82.1)		1.15 (1.00-1.31)		.05	1.06 (0.90-1.24)	.48
Stunting (< −2.00 SD)							
No	208/247 (84.2)		1 [Reference]		NA	1 [Reference]	NA
Yes	8/16 (50.0)		0.57 (0.28-1.15)		.12	0.74 (0.34-1.61)	.45
24 mo							
LAZ per 1.00 SD	214/257 (83.3)		1.20 (1.04-1.40)		.01	1.08 (0.91-1.28)	.39
Stunting (< −2.00 SD)							
No	195/228 (85.5)		1 [Reference]		NA	1 [Reference]	NA
Yes	19/29 (65.5)		0.72 (0.45-1.16)		.18	1.14 (0.67-1.95)	.62
**BMI at critical time points during infancy and childhood**^**c**^							
3 mo							
BAZ per 1.00 SD	201/244 (82.4)		1.08 (0.95-1.23)		.26	1.07 (0.92-1.23)	.40
Overweight (>2.00 SD)							
No	158/198 (79.8)		1 [Reference]		NA	1 [Reference]	NA
Yes	43/46 (93.5)		1.14 (0.81-1.60)		.44	1.03 (0.71-1.49)	.87
6 mo							
BAZ per 1.00 SD	206/250 (82.4)		1.08 (0.95-1.22)		.26	1.04 (0.90-1.20)	.57
Overweight (>2.00 SD)							
No	152/188 (80.9)		1 [Reference]		NA	1 [Reference]	NA
Yes	54/62 (87.1)		1.20 (0.88-1.64)		.25	1.15 (0.82-1.62)	.42
12 mo							
BAZ per 1.00 SD	216/262 (82.4)		1.14 (1.00-1.30)		.04	1.12 (0.97-1.30)	.11
Overweight (>2.00 SD)							
No	182/222 (82.0)		1 [Reference]		NA	1 [Reference]	NA
Yes	34/40 (85.0)		1.19 (0.83-1.72)		.35	1.18 (0.79-1.76)	.41
24 mo							
BAZ per 1.00 SD	212/255 (83.1)		1.10 (0.96-1.27)		.18	1.09 (0.94-1.27)	.25
Overweight (>2.00 SD)							
No	181/218 (83.0)		1 [Reference]		NA	1 [Reference]	NA
Yes	31/37 (83.8)		1.23 (0.84-1.81)		.29	1.16 (0.77-1.74)	.47

Abbreviations: BAZ, BMI-for-age *z* score; BMI, body mass index (calculated as weight in kilograms divided by square of height in meters); HR, hazard ratio; LAZ, length-for-age *z* score; NA, not applicable; WAZ, weight-for-age *z* score.

^a^ Denominators may differ owing to missing values.

^b^ Adjusted by small for gestational age, parental age, parental occupation, parental educational attainment, parity, maternal age at menarche, maternal BMI, household wealth index, and randomly assigned prenatal supplement regimens.

^c^ The BAZ at birth is not calculated by International Fetal and Newborn Growth Consortium for the 21st Century standards.

In addition, we examined the associations of underweight, stunting, and overweight with adolescent puberty onset. Similarly, the results showed that adolescent girls who experienced malnutrition during infancy had lower likelihood of entering puberty (adjusted HR, 0.51; 95% CI, 0.24-1.04), but the difference did not reach statistical significance in the multivariable analyses.

### Infant Rapid Growth and Adolescent Puberty Onset

A total of 109 of 254 infants (42.9%) showed rapid weight gain from birth to 24 months of age; 133 of 246 (54.1%), from birth to 3 months of age; 153 of 249 (61.4%), from birth to 6 months of age; 26 of 243 (10.7%), from 6 to 12 months of age; and 19 of 251 (7.6%), from 12 to 24 months of age. However, only rapid weight gain from birth to 24 months of age and birth to 3 months of age achieved a significant association with an increased likelihood of puberty onset among adolescent girls, with adjusted HRs of 1.40 (95% CI, 1.01-1.93) and 1.39 (95% CI, 1.02-1.91), respectively (Table 3).

**Table 3.** Association Between Infant Rapid Growth and Puberty Onset

Growth metric by age	Participants with puberty onset, No./total No. (%)^**a**^	Unadjusted		Adjusted^**b**^	
		HR (95% CI)	*P* value	HR (95% CI)	*P* value
**Rapid weight gain**					
Birth to 3 mo					
No	83/113 (73.5)	1 [Reference]	NA	1 [Reference]	NA
Yes	118/133 (88.7)	1.37 (1.03-1.81)	.03	1.39 (1.02-1.91)	.04
Birth to 6 mo					
No	75/96 (78.1)	1 [Reference]	NA	1 [Reference]	NA
Yes	129/153 (84.3)	1.15 (0.86-1.53)	.34	1.10 (0.79-1.52)	.58
6 to 12 mo					
No	178/217 (82.0)	1 [Reference]	NA	1 [Reference]	NA
Yes	22/26 (84.6)	1.08 (0.69-1.69)	.73	1.01 (0.63-1.62)	.98
12 to 24 mo					
No	193/232 (83.2)	1 [Reference]	NA	1 [Reference]	NA
Yes	15/19 (78.9)	0.99 (0.58-1.67)	.97	0.98 (0.55-1.75)	.94
Birth to 24 mo					
No	115/145 (79.3)	1 [Reference]	NA	1 [Reference]	NA
Yes	95/109 (87.2)	1.33 (1.02-1.75)	.04	1.40 (1.01-1.93)	.04
**Rapid length gain**					
Birth to 3 mo					
No	159/196 (81.1)	1 [Reference]	NA	1 [Reference]	NA
Yes	41/49 (83.7)	1.10 (0.78-1.55)	.58	1.18 (0.81-1.73)	.39
Birth to 6 mo					
No	164/199 (82.4)	1 [Reference]	NA	1 [Reference]	NA
Yes	40/48 (83.3)	1.09 (0.77-1.54)	.63	1.06 (0.72-1.55)	.78
6 to 12 mo					
No	132/164 (80.5)	1 [Reference]	NA	1 [Reference]	NA
Yes	68/79 (86.1)	1.00 (0.74-1.34)	.97	1.05 (0.76-1.45)	.76
12 to 24 mo					
No	187/226 (82.7)	1 [Reference]	NA	1 [Reference]	NA
Yes	23/27 (85.2)	1.10 (0.71-1.69)	.67	1.05 (0.66-1.68)	.83
Birth to 24 mo					
No	168/200 (84.0)	1 [Reference]	NA	1 [Reference]	NA
Yes	43/53 (81.1)	1.02 (0.73-1.43)	.91	1.01 (0.69-1.48)	.95

Abbreviations: HR, hazard ratio; NA, not applicable.

^a^ Denominators may differ owing to missing values.

^b^ Adjusted by small for gestational age, parental age, parental occupation, parental educational attainment, parity, maternal age at menarche, maternal BMI, household wealth index, and randomized prenatal supplement regimens.

In addition, 53 of 253 infants (20.9%) showed rapid length gain from birth to 24 months of age; 49 of 245 (20.0%), from birth to 3 months of age; 48 of 247 (19.4), from birth to 6 months of age; 79 of 243 (32.5%), from 6 to 12 months of age; and 27 to 253 (10.7%), from 12 to 24 months of age. We did not find that rapid length gain during the aforementioned infancy periods was associated with adolescent puberty onset. Similar results were observed in unadjusted models (Table 3).

## Discussion

In this prospective birth cohort study of girls conducted in rural western China, we found that the median age of puberty onset was 11 (IQR, 10-11) years. A higher WAZ during infancy was associated with earlier puberty onset. Furthermore, rapid weight gain in infancy during the first 2 years of life, particularly from birth to 3 months of age, was associated with an increased likelihood of earlier puberty onset. However, we did not find that length-related indicators during early life were associated with adolescent puberty onset, such as LAZ, stunting, and rapid length gain. Similar null associations were observed for birth weight and LAZ.

In the present study, 241 girls (82.0%) experienced puberty onset at a median age of 11 (IQR, 10-11) years. Secular trend analyses showed that the median age at puberty onset in Chinese girls declined from 9.8 years in 1993 to 9.2 years in 2010.^25,26^ However, most of the study areas were located in metropolises such as Shanghai and Guangzhou and did not include rural western areas,^25^ where malnutrition is still relatively prevalent.^27^ In western China, only 1 study conducted in 2001-2002 in Shaanxi Province observed puberty onset and reported a mean age of 12.1 years,^28^ which was 1 year older than our result. This discrepancy was expected given the improvement of nutritional status among children in western China during the last decade.^29^ However, the earlier puberty onset observed in developed countries warrants other explanations beyond undernutrition. Crocker et al^30^ reported that the secular change in age at puberty onset parallels the increasing prevalence of overweight and obesity in the United States. Similar changes have been observed in Western Europe.^7^ Consequently, we examined the associations of infancy growth in early life with adolescent puberty onset in an attempt to understand the mechanism behind these links.

Consistent with the Avon Longitudinal Study in British girls,^31^ we did not find any associations between birth size and timing of puberty onset. Most studies,^8,32,33^ however, found that intrauterine growth restriction and lower birth weight were associated with earlier puberty onset. In contrast, some studies^34^ have reported that girls with higher birth weights tend to have earlier puberty onset. The divergent results may partly be explained by participants from different ethnicities and the different analytical approaches and birth weight measures used.

To the best of our knowledge, our study is the first to fully and comparatively assess the roles of infant WAZ, LAZ, and BAZ during the first 2 years of puberty onset. We found that higher WAZ was associated with earlier puberty onset, and girls who experienced underweight in early life were less likely to enter puberty. In line with prior studies, which used menarche to assess puberty onset,^13,14,35^ we also found that infant rapid weight gain was associated with an increased likelihood of puberty onset. Although the biological mechanisms are not yet fully illustrated, studies speculate that rapid weight gain in early life has been linked to elevated insulinlike growth factor 1 concentrations and insulin resistance, adrenal androgen concentrations, and risk of obesity, all of which could promote the activity of the gonadotropin-releasing hormone pulse generator, consequently influencing the timing of puberty.^14^

In addition, our results (Table 3) show that rapid weight gain from birth to 3 months of age plays a key role in influencing the timing of puberty onset. A study from Jamaica that used different growth indicators and analytical approaches^36^ reported that the rapid weight gain from birth to 6 months of age was associated with earlier puberty onset. Similar results have been reported in other studies with longer intervals, from birth to 12 months of age in the Danish National Birth Cohort^32^ and from birth to 20 months of age in a British study.^31^ The lack of association in our study could be explained by the sample size and different ethnicities. Furthermore, in these 2 studies, the information on Tanner stage and weight was self-reported. Inconsistent with our null findings, a study conducted in North Carolina^34^ reported that girls with greater early weight gain from 6 to 12 months of age and from 12 to 24 months of age reached advanced Tanner stages at earlier ages, which may be due to the small sample size. Our results suggest for the first time that rapid weight gain within the first 3 months of age plays an independent role in subsequent pubertal development, and postnatal growth should be monitored closely as soon as possible after birth. In addition, some twin analyses have reported that weight gain is less likely to be influenced by genetic background during infancy than during childhood, suggesting a susceptible, modifiable window of infancy for long-term health outcomes.^37,38^ Interventions designed to reduce infant rapid weight gain through early nutrition management and maintain a healthy weight before, during, and after pregnancy may be feasible and practical.^39,40^

We did not find that length gain was associated with puberty onset in the present study. We hypothesize that this null finding may be due to the small number of participants who experienced rapid length gain, ranging from 10.7% to 32.5%. However, a British cohort study reported that rapid height changes in infancy, particularly from 4 to 12 months of age, may be related to later age at menarche.^41^ The difference in findings suggests complex pathways that should be replicated in larger studies. We could not rule out the possibility that there is no association between length gain during infancy and puberty onset. Woo and colleagues^42^ reported that faster linear growth was associated with higher fat-free mass and lower percentage of body fat later in life, which may support our results, because body fat stores but not fat-free mass influence estradiol level. Thus, although linear growth in early life has been shown to be associated with many subsequent health outcomes, such as cognitive and motor development,^43^ puberty onset may not be one of them.

To our knowledge, our study is the first to investigate the influence of infancy growth on early puberty onset in girls in rural western China. The Tanner stages we used to determine the onset of puberty are considered the standard criteria by which prepubertal and pubertal girls can be reliably distinguished.^44^ This is particularly critical for girls who are in early puberty and are slightly overweight or obese, which may falsely suggest the presence of breast development with the staging of thelarche by visual inspection only.

### Limitations

The study has a few limitations. First, our study was conducted within the context of a trial of antenatal micronutrient supplementation, which may result in selection bias, limiting the generalizability of our results. In addition, 253 of 547 participants (46.3%) were lost to follow-up, although most of the baseline characteristics were balanced between participants who were lost to follow-up and those who were followed up. Third, age at puberty onset for girls with Tanner stages of greater than 2 was self-reported, which may involve the risk of recall bias. However, given the importance of puberty onset for girls and the short time after experiencing this milestone event, we considered the risk of recall bias to be acceptable. Interstaff variability in anthropometry measurements exists during different visits. Finally, we did not include all possible covariates, such as maternal smoking during pregnancy, the type of feeding, excessive gestational weight gain, and stressful life events experienced during pregnancy. Future studies with strict designs are warranted to investigate the causal association between weight gain in infancy and puberty onset.

## Conclusions

In this prospective cohort study, girls who experienced rapid weight gain and had higher WAZ during the first 2 years of life had a higher likelihood of early onset of puberty. We identified the period of the first 3 months after birth as a critical window for the correlative role of weight-related indicators in puberty onset, indicating the necessity of monitoring weight growth as soon as possible after birth. Further research is needed to investigate whether these associations are causal and whether weight growth in early life could be modified to lower subsequent disease risk.

## Notes

Supplement. **eTable.** Comparison of Baseline Characteristics of the Adolescents Followed Up and Lost to Follow-up **eFigure.** Participant Flowchart
