# Supplementary material for: Association of Infant Physical Development and Rapid Growth With Pubertal Onset Among Girls in Rural China
Source: JAMA Netw Open. 2021 May 3;4(5):e216831. doi: 10.1001/jamanetworkopen.2021.6831 (PMC8094009; doi:10.1001/jamanetworkopen.2021.6831)
Supplement: Supplement. — eTable. Comparison of Baseline Characteristics of the Adolescents Followed Up and Lost to Follow-up eFigure. Participant Flowchart [file jamanetwopen-e216831-s001.pdf]

Supplementary Online Content

Wei J, Liu S, Cheng Y, Yang W, Zhu Z, Zeng L. Association of infant physical development and rapid growth with pubertal onset among girls in rural China. *JAMA Netw Open*. 2021;4(5):e216831. doi:10.1001/jamanetworkopen.2021.6831

eTable. Comparison of Baseline Characteristics of the Adolescents Followed Up and Lost to Follow-up

eFigure. Participant Flowchart

This supplementary material has been provided by the authors to give readers additional information about their work.

© 2021 Wei J et al. *JAMA Network Open*.

eTable. Comparison of Baseline Characteristics of the Adolescents Followed Up and Lost to Follow-up

	Follow-up/ n (%)	Lost to follow-up/ n (%)	*P* value[a]
Total number	294(53.8)	253(46.2)	
**Parental characteristics in pregnancy**			
Maternal age (years) (Mean ± standard deviation)	23.43(4.09)	23.71(4.05)	0.42
Maternal education			
< 3 years	12(4.1)	5(2.0)	0.06
Primary	59(20.1)	48(19.0)	
Secondary	168(57.3)	169(66.8)	
High school+	54(18.5)	31(12.2)	
Maternal occupation			
Farmer	237(81.0)	210(83.3)	0.46
Others	56(19.0)	42(16.7)	
Parity			
0	231(78.6)	194(76.7)	0.02
1	49(16.7)	56(22.1)	
≥ 2	14(4.7)	3(1.2)	
Randomized regimens			
Folic acid	99(33.7)	84(33.2)	0.82
Iron/Folic acid	94(32.0)	87(34.4)	
Multiple micronutrients	101(34.3)	82(32.4)	
Paternal age (years) (Mean ± standard deviation)	26.71(3.96)	27.13(3.98)	0.21
Paternal education			
< 3 years	3(1.0)	0(0.0)	0.29
Primary	21(7.1)	18(7.2)	
Secondary	190(64.6)	174(69.3)	
High school+	80(27.3)	59(23.5)	
Paternal occupation			
Farmer	207(70.4)	189(74.7)	0.26
Others	87(29.6)	64(25.3)	
Household wealth index			
Low	71(24.2)	57(22.5)	0.90
Middle	110(37.4)	96(37.9)	
High	113(38.4)	100(39.6)	
**Infant**			
Birth weight(g) (Mean ± standard deviation)	3094(380)	3058(375)	0.26
Birth length(cm) (Mean ± standard deviation)	48.9(2.3)	48.7(2.4)	0.38
Gestational weeks at delivery (Mean ± standard deviation)	40(1)	40(2)	0.87

© 2021 Wei J et al. *JAMA Network Open*.

	Follow-up/ n (%)	Lost to follow-up/ n (%)	*P* value[a]
Small for gestational age (< 10th population percentile)			
No	236(82.5)	198(80.5)	0.55
Yes	50(17.5)	48(19.5)	
Low birth weight (<2500g)			
No	279(95.2)	238(95.6)	0.84
Yes	14(4.8)	11(4.4)	
Preterm birth (<37 weeks)			
No	287(97.6)	242(95.6)	0.20
Yes	7(2.4)	11(4.4)	

[a] Comparisons were conducted using analysis of t test for continuous variables and $\chi 2$ test for categorical variables, respectively.

© 2021 Wei J et al. *JAMA Network Open*.

eFigure. Participant Flowchart

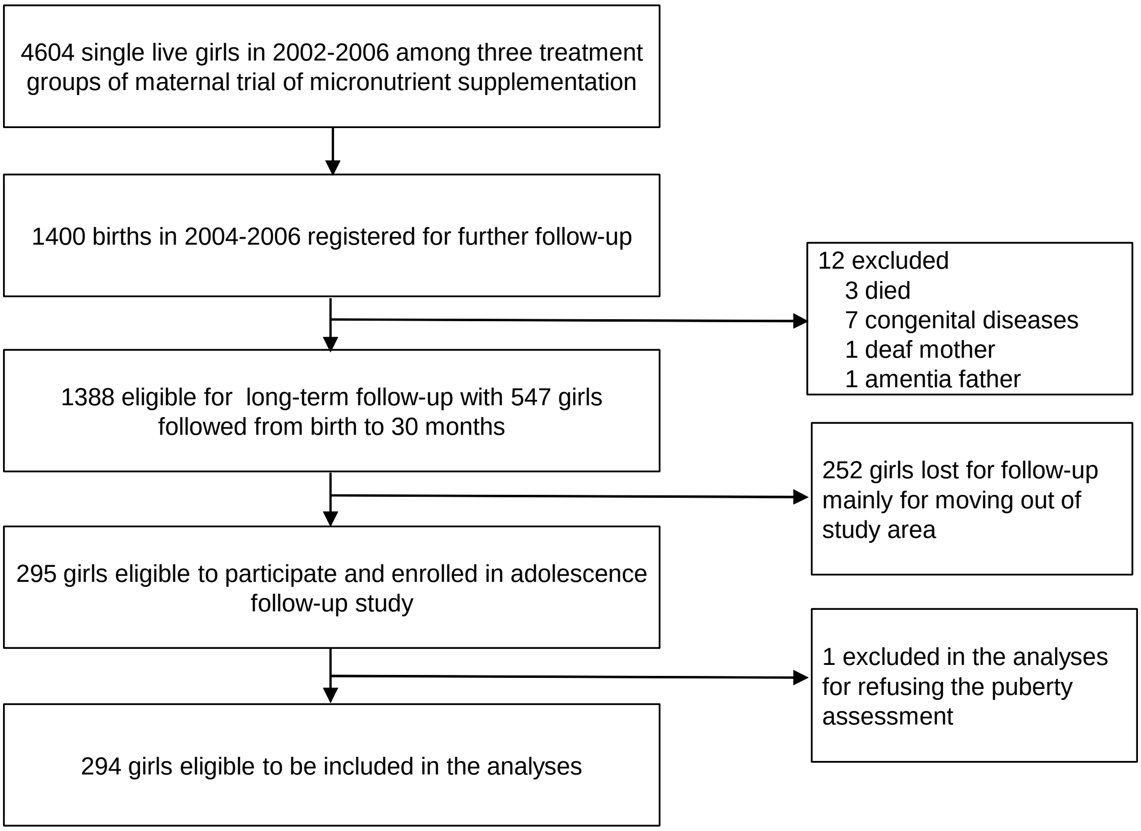

© 2021 Wei J et al. *JAMA Network Open*.
